# OsMS188 Is a Key Regulator of Tapetum Development and Sporopollenin Synthesis in Rice

**DOI:** 10.1186/s12284-020-00451-y

**Published:** 2021-01-06

**Authors:** Yu Han, Si-Da Zhou, Jiong-Jiong Fan, Lei Zhou, Qiang-Sheng Shi, Yan-Fei Zhang, Xing-Lu Liu, Xing Chen, Jun Zhu, Zhong-Nan Yang

**Affiliations:** Shanghai Key Laboratory of Plant Molecular Sciences, College of Life Sciences, Shanghai Normal University, 100 Guilin Road, Shanghai, 200234 China

**Keywords:** Rice, Tapetum, Pollen sexine, Sporopollenin, Transcription factor

## Abstract

**Background:**

During anther development, the tapetum provides essential nutrients and materials for pollen development. In rice, multiple transcription factors and enzymes essential for tapetum development and pollen wall formation have been cloned from male-sterile lines.

**Results:**

In this study, we obtained several lines in which the MYB transcription factor *OsMS188* was knocked out through the CRISPR-Cas9 approach. The *osms188* lines exhibited a male-sterile phenotype with aberrant development and degeneration of tapetal cells, absence of the sexine layer and defective anther cuticles. *CYP703A3*, *CYP704B2*, *OsPKS1*, *OsPKS2*, *DPW* and *ABCG15* are sporopollenin synthesis and transport-related genes in rice. Plants with mutations in these genes are male sterile, with a defective sexine layer and anther cuticle. Further biochemical assays demonstrated that OsMS188 binds directly to the promoters of these genes to regulate their expression. *UDT1*, *OsTDF1*, *TDR*, *bHLH142* and *EAT1* are upstream regulators of rice tapetum development. Electrophoretic mobility shift assays (EMSAs) and activation assays revealed that TDR directly regulates *OsMS188* expression. Additionally, protein interaction assays indicated that TDR interacts with OsMS188 to regulate downstream gene expression.

**Conclusion:**

Overall, OsMS188 is a key regulator of tapetum development and pollen wall formation. The gene regulatory network established in this work may facilitate future investigations of fertility regulation in rice and in other crop species.

**Supplementary Information:**

The online version contains supplementary material available at 10.1186/s12284-020-00451-y.

## Background

Rice (*Oryza sativa* L.) is one of the world’s most important agricultural crop species and supports the nutritional requirements of more than half of the global population. Heterosis from hybrid breeding significantly increases agricultural yields of rice and other crop species (Chen and Liu 2014). The use of male-sterile plants, which serve as necessary breeding materials, constitutes a convenient approach for producing hybrid varieties. A large number of male-sterile lines of rice have been identified to date, and related genes have been cloned. Most of these genes play essential roles in tapetum development and pollen formation.

The tapetum is the innermost layer of the four sporophytic layers of the anther wall. The tapetal layer is in directly contact with developing gametophytes and provides necessary materials and nutrients for microspore development (Mariani et al. 1990; McCormick 1993; Ariizumi and Toriyama 2011). Several transcription factors essential for tapetum development have been cloned from male-sterile lines of rice. *Undeveloped Tapetum1* (*UDT1*) (Jung et al. 2005), *Tapetum Degeneration Retardation* (*TDR*) (Li et al. 2006), *ETERNAL TAPETUM 1/DELAYED TAPETUM DEGENERATION* (*EAT1/DTD*) (Niu et al. 2013; Ji et al. 2013) and *INTERACTING PROTEIN 2*/*bHLH142* (*TIP2/bHLH142*) (Fu et al. 2014; Ko et al. 2014) encode transcription factors of the bHLH family. *BHLH142* acts downstream of *UDT1* but upstream of *TDR* and *EAT1* in tapetum development (Fu et al. 2014; Ko et al. 2014). *OsTDF1* encodes an R2R3-MYB-family protein (Cai et al. 2015). These genes play an essential role in early tapetum development in rice. Plants with mutations in these transcription factors exhibit a similar phenotype in which abnormally vacuolated and enlarged tapetal cells occupy the locule space during anther development. *PTC1* encodes a PHD-finger protein that functions in the late stage of tapetum development, at which time the anther locule forms in the mutants (Li et al. 2011).

The tapetum directly provides materials for pollen wall formation and nutrients for pollen development. Several tapetum genes involved in pollen wall formation have been cloned from male-sterile lines of rice. CYP703A3 and CYP704B2 belong to the cytochrome P450 family and catalyse the in-chain 7-hydroxylation of lauric acid and the ω-hydroxylation of fatty acids with 16 and 18 carbon chains, respectively (Li et al. 2010; Yang et al. 2014). OsPKS1 and OsPKS2 condense fatty acyl-CoA into triketide and tetraketide a-pyrones, respectively, which are predicted to be components of the sporopollenin precursor (Shi et al. 2018; Zou et al. 2018). *OsACOS12* encodes an acyl-CoA synthetase that esterifies fatty acids to fatty acyl-CoA in the plastids of the tapetum (Li et al. 2016; Yang et al. 2017). *DPW* encodes a fatty acid reductase that converts the palmitoyl-acyl carrier protein to fatty alcohols, producing sporopollenin monomers (Shi et al. 2011). The resultant sporopollenin precursors are predicted to be transported to anther locules by the membrane transport protein ABCG15 (Qin et al. 2013). Because the above genes are expressed in the tapetum, elucidation of the regulatory mechanism involving tapetal transcription factors and the expression of pollen wall-related genes is important for understanding pollen formation. A previous study proposed that the expression of several sporopollenin-related genes, such as *CYP704B2* and *CYP703A3,* was regulated by TDR in rice, which is considered a key transcriptional regulator in pollen wall formation (Shi et al. 2015). However, the rice tapetal genetic pathway includes other transcription factors. The mechanisms of these regulators and downstream targets in pollen wall formation require further genetic and biochemical validation.

A previous investigation showed that the knockdown of *OsMS188* (LOC_Os04g39470), which encodes a MYB transcription factor in rice, resulted in defective pollen formation and reduced fertility (Zhang et al. 2010). Here, we obtained *OsMS188* knockout lines through the CRISPR-Cas9 approach; these lines were completely male sterile. The *osms188* mutants presented aberrant vacuolized tapetal cells, an absent sexine layer and a defective anther cuticle. The expression of multiple sporopollenin genes is directly regulated by OsMS188. Therefore, OsMS188 is a key regulator of tapetum development and pollen wall and cuticle layer formation in rice. Additionally, *OsMS188* is directly regulated by and interacts with TDR to activate the expression of downstream targets, which extends the tapetal regulatory network. The elucidation of this genetic pathway not only is helpful for understanding anther development but also can provide clues for further investigation of fertility regulation.

## Results

### Knockout of *OsMS188* Causes Complete Male Sterility

**Fig. 1:**
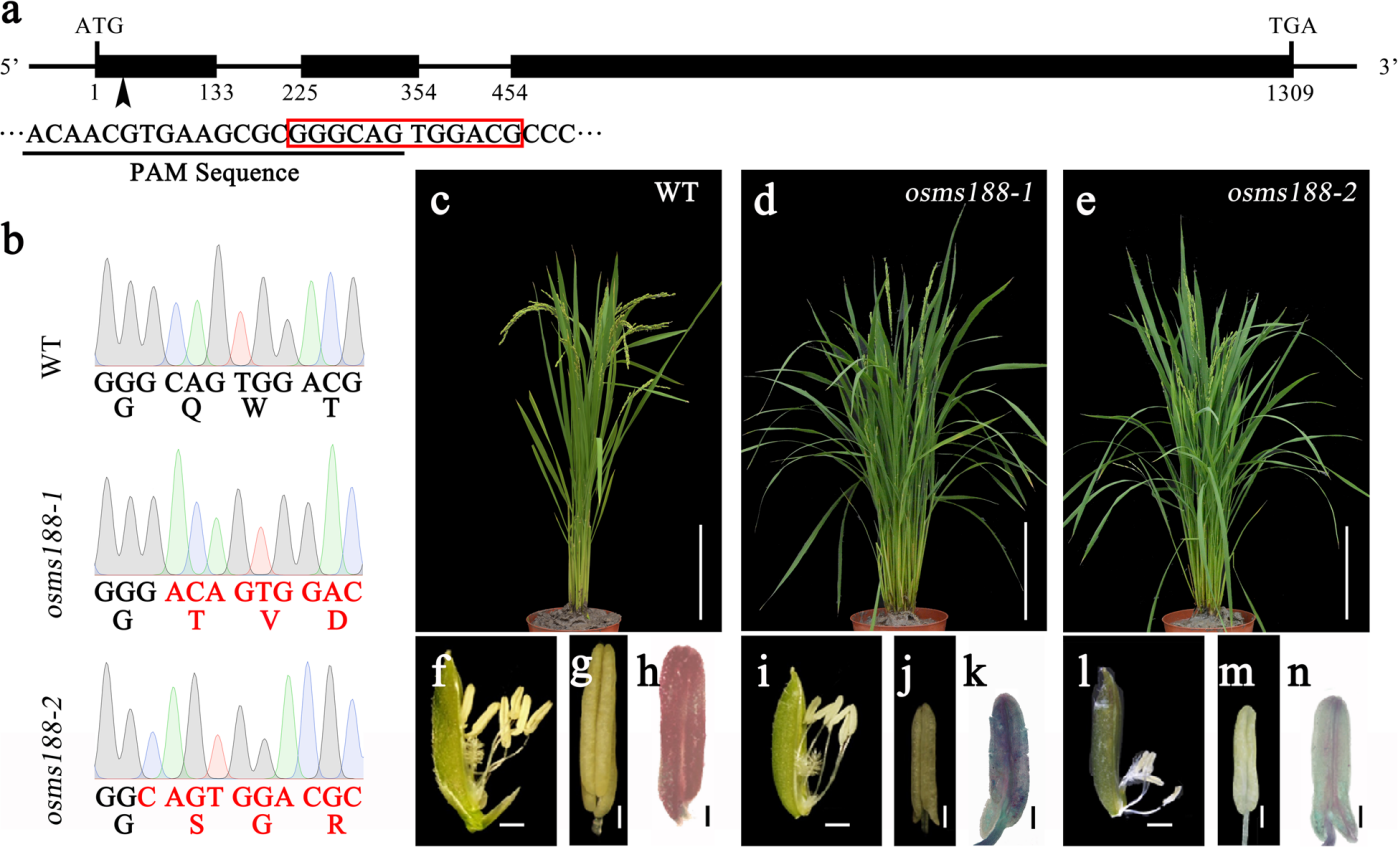
Mutation of *OsMS188* to obtain complete male-sterile mutants. **a** Gene structure and the editing site of *OsMS188*. The black box indicates the exon. The black arrow indicates the site of the PAM sequence. The red box around the sequence shows the mutation sites in **b**. **b** Mutation sites of *OsMS188* in the wild type (WT), *osms188–1* and *osms188–2*; the red font indicates the variation in nucleic acids and amino acids. **c**-**e** Phenotype of WT plant (**c**), *osms188–1* mutant (**d**) and *osms188–2* mutant (**e**). **f**-**n** Spikelets, mature anthers and Alexander staining of the WT plant (**f**-**h**), *osms188–1* mutant (**i**-**k**) and *osms188–2* mutant (**l**-**n**). Bars: (**c**-**e**) 20 cm; (**f**, **i**, **l**) 1 mm; (**g**-**h**, **j**-**k**, **m**-**n**) 200 μm

A knockout mutant of *OsMS188* was obtained via the CRISPR-Cas9 approach in which the first exon of *OsMS188* (LOC_Os04g39470) in wild type Nipponbare was targeted (Fig. 1a). A total of 26 transgenic lines were obtained. Among these transgenic lines, two lines exhibiting an adenosine insertion (*osms188–1*) and a guanine deletion (*osms188–2*) in their first exons were identified. These mutations led to a frameshift of *OsMS188* with a premature stop codon in these two lines (Fig. 1b). Compared with the fertile phenotype of the wild type (Fig. 1c, f and g), the phenotype of both transgenic lines was male sterile, and the plants had white, shrunken anthers (Fig. 1d, i, j, e, l and m). Alexander staining showed that the *osms188* anthers contained no mature pollen grains and presented only a few degenerated pollen remnants (Fig. 1h, k and n). To confirm whether the male-sterile phenotype of the transgenic lines was caused by the mutation of *OsMS188*, we crossed these lines with the wild type. The F1 plants showed normal fertility, and the segregation ratio of the fertile and sterile phenotype in the F2 generation was approximately 3:1 (202:82, χ^2^_3:1_=2.0704). We sequenced the *OsMS188* locus in the male-sterile lines in the F2 population. This locus had a mutation in all the lines. Taken together, these results demonstrate that the target mutations of *OsMS188* caused male sterility in the transgenic lines.

### The Sexine Layer Is Absent in the *osms188* Mutant

**Fig. 2:**
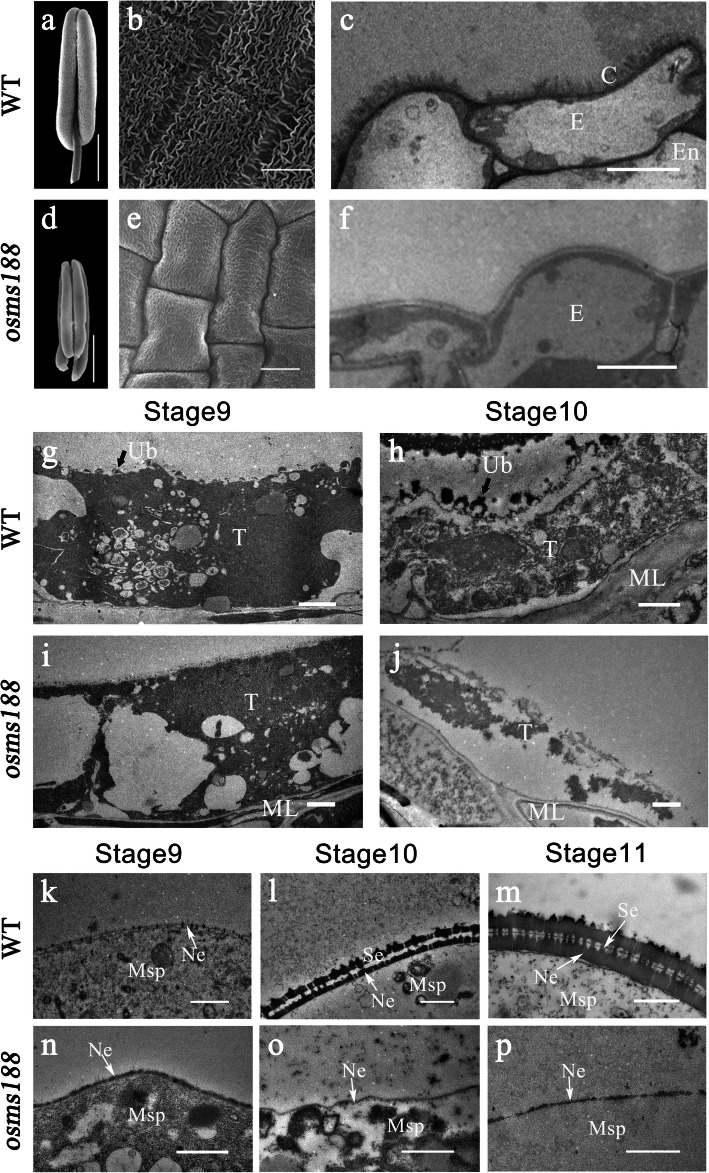
Electron micrographs of anthers and pollen grains of the WT and *osms188* mutant. SEM observations showing the anther and pollen development of the WT (**a**-**b**) and *osms188* (**d**-**e**). Anther morphology of the WT (**a**) and *osms188* (**d**) and the epidermal surfaces of WT (**b**) and *osms188* (**e**) anthers. TEM observations of tapetum development and sexine formation of the WT (**c**, **g**, **h**, **k**, **l**, **m**) and *osms188* (**f**, **i**, **j**, **n**, **o**, **p**). Cuticle layer of the WT (**c**) and *osms188* (**f**). The tapetal cells of the WT (**g**) and *osms188* (**i**) at stage 9 and the tapetal cells of the WT (**h**) and *osms188* (**j**) at stage 10. Pollen wall formation in the WT (**k**, **l**, **m**) and *osms188* (**n**, **o**, **p**) during stages 9–11. C, cuticle; E, epidermis; En, endothecium; ML, middle layer; Msp, microspore; Ne, nexine; Se, sexine; T, tapetum; Ub, Ubisch body. Bars: (**a**, **d**) 500 μm; (**b**, **e**) 20 μm; (**c**, **f**) 5 μm; (**g**-**l**, **o**-**p**) 1 μm; (**m**, **n**) 0.5 μm

To identify the specific defect in pollen development, cytological analyses were performed. Scanning electron microscopy (SEM) showed that the anthers of *osms188* shrank and were much smaller than those of the wild type (Fig. 2a and d). The surface of the wild-type anthers was covered with a normal cuticle layer (Fig. 2b). However, the reticulate cuticle layer was absent on the surface of the anthers of *osms188* (Fig. 2e), which is consistent with the characteristics of other sporopollenin-related mutants (Li et al. 2010; Shi et al. 2011; Yang et al. 2014; Li et al. 2016). In rice, anther development is divided into 14 stages according to morphological characteristics (Zhang and Wilson 2009). Semi-thin sections showed that no detectable differences were observed between the *osms188* and wild type plants in terms of anther development before stage 8. However, the tapetal cells became irregularly vacuolated after stage 8 (Fig. S1). To illustrate the detailed defect in pollen development in this mutant, we performed transmission electron microscopy (TEM). The results showed that the reticulate cuticle layer on the anther surface in *osms188* was obviously thinner than that in the wild type (Fig. 2c and f). At stage 9, TEM showed that Ubisch bodies initially presented on the internal surface of the wild type tapetum (Fig. 2g), and probacular materials were deposited outside the microspores (Fig. 2k). At stage 10, abundant Ubisch bodies had been clearly secreted on the internal surface of the tapetum (Fig. 2h). Additionally, the sexine layer of the pollen wall with tectum and baculum structures gradually formed in the wild type (Fig. 2l and m). However, at stage 9, the tapetal cytoplasm of os*ms188* was filled with large vacuoles without Ubisch bodies, and probacular materials were not observed on the surface of *osms188* microspores (Fig. 2i and n). During the late stages, the tapetal protoplasts of *osms188* were largely degenerated, and no Ubisch bodies accumulated (Fig. 2j). Most developing microspores were also degraded in the *osms188* mutant, and only a few microspore residues remained. The sexine layer was completely absent on the surface of these residues (Fig. 2o and p).

### The PCD Process Is Altered in the Tapetal Cells of the *osms188* Mutant

**Fig. 3:**
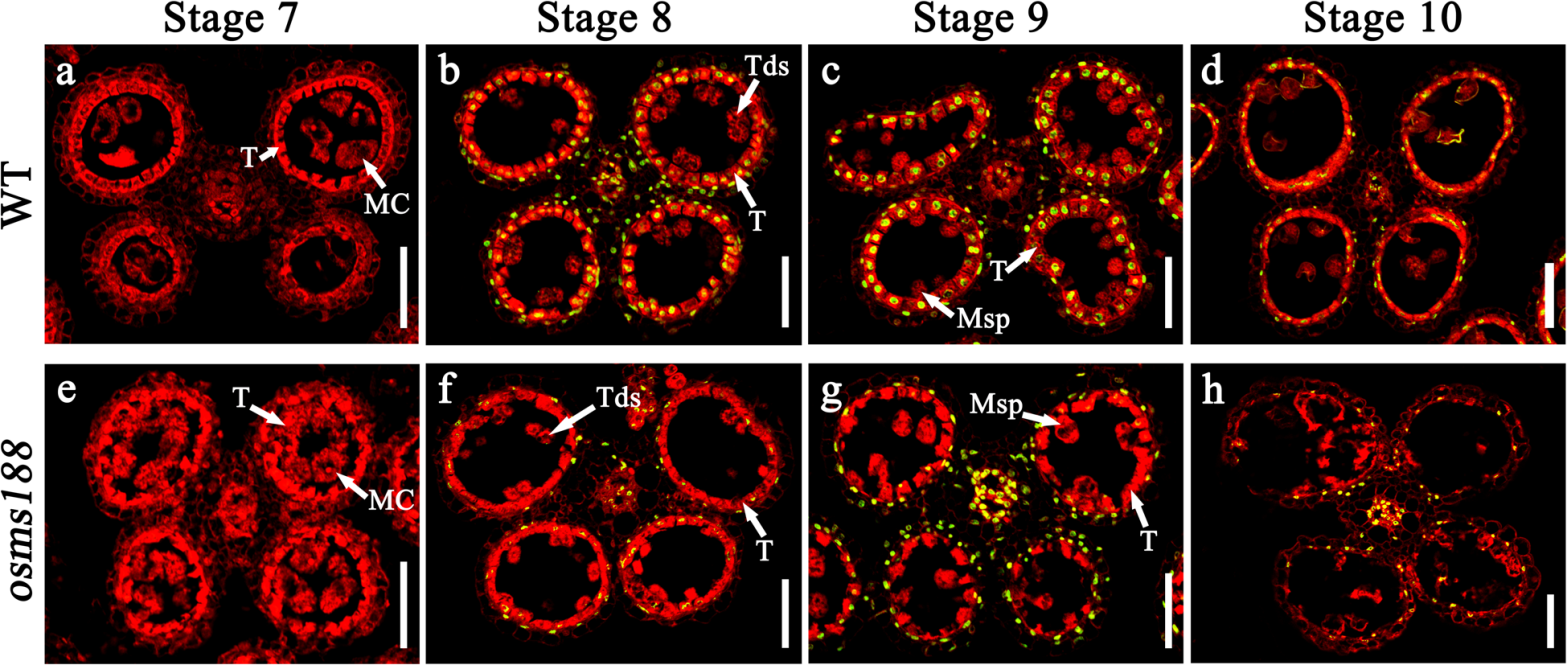
TUNEL analysis in the anthers of WT and *osms188*. The TUNEL signals are indicated with green fluorescence. The red fluorescence indicates nuclei stained with propidium iodide. At stage 7, no TUNEL signals were detected in the anthers of the WT (**a**) or *osms188* (**e**). The TUNEL signals of the WT were normal at stage 8 (**b**), stage 9 (**c**) and stage 10 (**d**). Weak TUNEL signals were detected at stage 8 (**f**), stage 9 (**g**) and stage 10 (**h**) of the *osms188* anthers. Bars = 50 μm. MC, meiotic cell; T, tapetum; Tds, tetrads; Msp, microspore

The tapetum undergoes cellular degradation by programmed cell death (PCD) during the late stage of anther development. To understand whether tapetum PCD is affected in *osms188*, a TUNEL assay was performed. No fluorescence signals were observed in the anthers of either wild type or *osms188* plants during the meiosis stage (Fig. 3a and e). In the wild type, TUNEL signals were initially detected in tapetal cells at the tetrad stage (Fig. 3b), and their signals became stronger at the microspore release stage (Fig. 3c). At stage 10, the signals were still visible, although they were weaker than those in the previous two stages (Fig. 3d). In the *osms188* mutant, the TUNEL signals were very weak in the aberrant tapetal cells at all these stages (Fig. 3f, g and h). These observations demonstrate that tapetum PCD in *osms188* is obviously slower than that in the wild type, suggesting that OsMS188 is involved in regulating tapetum degeneration during anther development.

### *OsMS188* Is Highly Expressed in Tapetal Cells during Anther Development

**Fig. 4:**
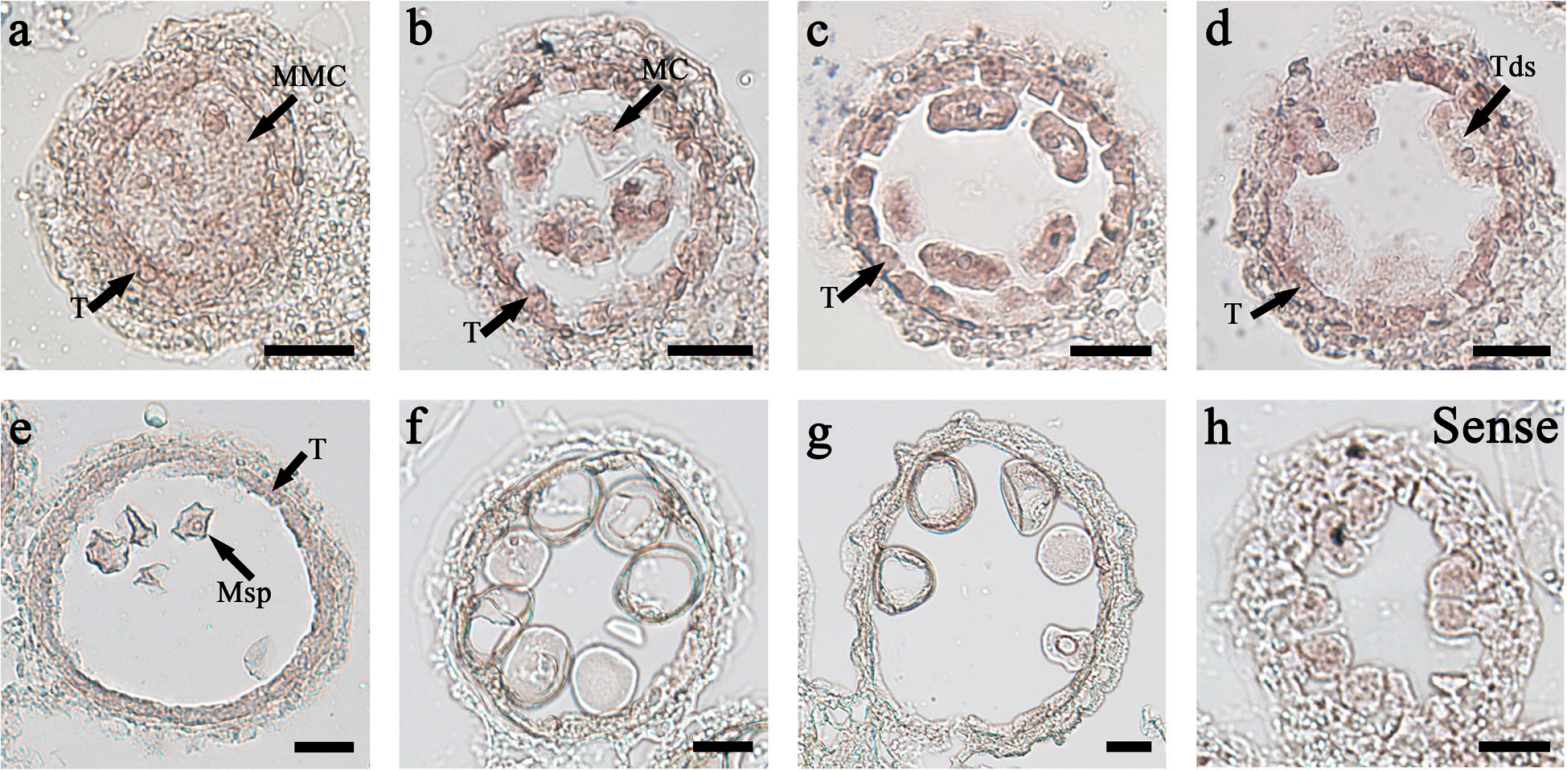
In situ analysis of *OsMS188* expression in wild-type anthers at different developmental stages. In situ hybridization of OsMS188 transcripts at stage 6 (**a**), stage 7 (**b**), stage 8a (**c**), stage 8b (**d**), stage 9 (**e**), stage 10 (**f**) and stage11 (**g**) anthers with an *OsMS188* antisense probe. The anthers at stage 8b (**h**) hybridized to an *OsMS188* sense probe. MMC, microspore mother cell; MC, meiotic cell; Msp, microspore; T, tapetum; Tds, tetrads. Bars = 20 μm

Cytological observations have revealed that *OsMS188* is essential for tapetum development and pollen wall formation in rice. RNA in situ hybridization was performd to understand *OsMS188* expression in the anthers in detail. Transcripts of *OsMS188* were initially detected at stage 6 (Fig. 4), and their abundance gradually increased in tapetal and meiocyte cells at stage 7 (Fig. 4b). At the tetrad stage, the *OsMS188* transcript abundance peaked in the tapetal cells (Fig. 4c and d). After microspore release, the expression of *OsMS188* radically decreased in the tapetum and microspores (Fig. 4e, f and g). In the control, the sense probe exhibited only background signals at the tetrad stage (Fig. 4h). The expression pattern of *OsMS188* in tapetal cells is in accordance with the timing of pollen wall formation.

### *TDR* Directly Regulates *OsMS188*

**Fig. 5:**
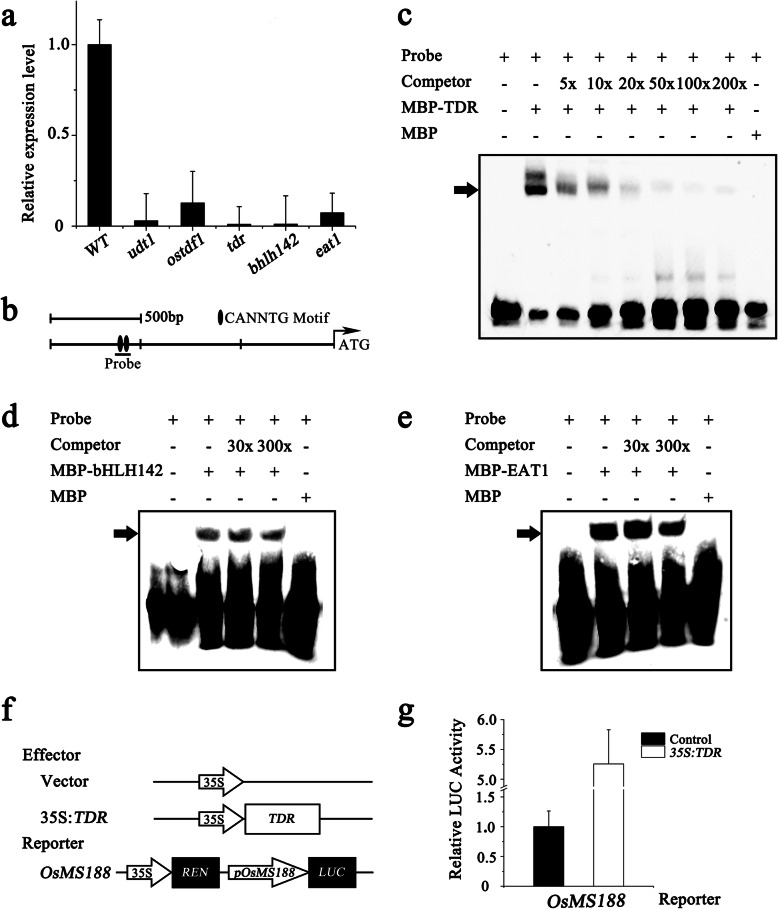
TDR directly binds and activates the expression of *OsMS188*. **a** QRT-PCR analysis of OsMS188 expression in the inflorescences of the WT, *udt1*, *ostdf1*, *tdr*, *bhlh142* and *eat1*. **b** Potential binding sites of bHLH transcription factors in the *OsMS188* promoter region. The black dots indicate the CANNTG motif. **c** EMSAs showing that *TDR* binds to the fragment of the *OsMS188* promoter region. The MBP-tagged *TDR* proteins were mixed with a biotin-labelled probe, and unlabelled probes were used as competitors. The arrowhead indicates a shifted band. **d**-**e** The abundance of the shifted band was not reduced by unlabelled competitor probes mixed with bHLH142 and EAT1. **f**-**g** Transient dual-luciferase transactivation of the *OsMS188* promoter by TDR in protoplasts. Three replicates were assessed, and the Y-axis shows the ratio of Luciferase/Renilla. The errors bar indicate the SDs

*UDT1*, *OsTDF1*, *TDR*, *bHLH142*, and *EAT1* encode transcription factors that are essential for rice tapetum development (Jung et al. 2005; Li et al. 2006; Niu et al. 2013; Ko et al. 2014; Cai et al. 2015). We analysed the relationship between *OsMS188* and these five transcription factors. Both RT-PCR and qRT-PCR analyses showed that *OsMS188* expression was significantly downregulated in all the plants with mutations in these genes (Fig. 5a; Fig. S2). Among these transcription factor-coding genes, *OsTDF1* encodes a MYB transcription factor, and the other four encode bHLH transcription factors. Since *UDT1* encodes an upstream regulator of *OsTDF1*, it is unlikely to be a direct regulator of *OsMS188*. Previous work showed that the expression of *OsMS188* was significantly reduced in *ostdf1* and that of *TDR* and *EAT1* was also reduced to some extent, indicating that these genes are downstream of *OsTDF1* (Cai et al. 2015). *TDR*, *bHLH142*, and *EAT1* encode bHLH transcription factors. We sought to determine which transcription factors could directly regulate the expression of *OsMS188*. The promoter region of *OsMS188* (− 1142 and − 1097) contains two core motifs of bHLH cis-elements (CANNTG) (Fig. 5b). The bHLH142, EAT1 and TDR proteins were expressed in and purified from *Escherichia coli* Rosetta. EMSAs showed that the recombinant TDR proteins could bind to DNA probes containing the ‘CANNTG’ core motif. Unlabelled probes competed for DNA binding when applied at a 5-, 10-, 20-, 50-, 100- or 200-fold concentration (Fig. 5c). However, the abundance of the shifted bands could not be reduced by unlabelled competitor probes when the bHLH142 and EAT1 proteins were incubated together with the probe for the *OsMS188* promoter (Fig. 5d and e). These data show that TDR, rather than bHLH142 or EAT1, directly binds to the promoter of *OsMS188* in vitro. A transient expression assay using Arabidopsis protoplasts was performed to determine whether TDR could activate the expression of *OsMS188*. We generated constructs containing the coding DNA sequence (CDS) of TDR driven by the cauliflower mosaic virus (CaMV35S) promoter (*p35s::TDRnos*) as an effector, while the firefly luciferase (LUC) reporter gene was driven by the *OsMS188* promoter together with the 35S::Renilla gene as a reporter (*pOsMS188::LUC*). A construct containing only the 35S promoter and the terminator was used as a negative control (*p35s::nos*). When *p35s::TDRnos* and *pOsMS188::LUC* were cotransformed into protoplasts, LUC luminescence significantly increased compared with the background level in the negative control (Fig. 5f and g). These results indicated that TDR directly binds the promoter of *OsMS188* to regulate its expression during anther development.

### OsMS188 Interacts with TDR

**Fig. 6:**
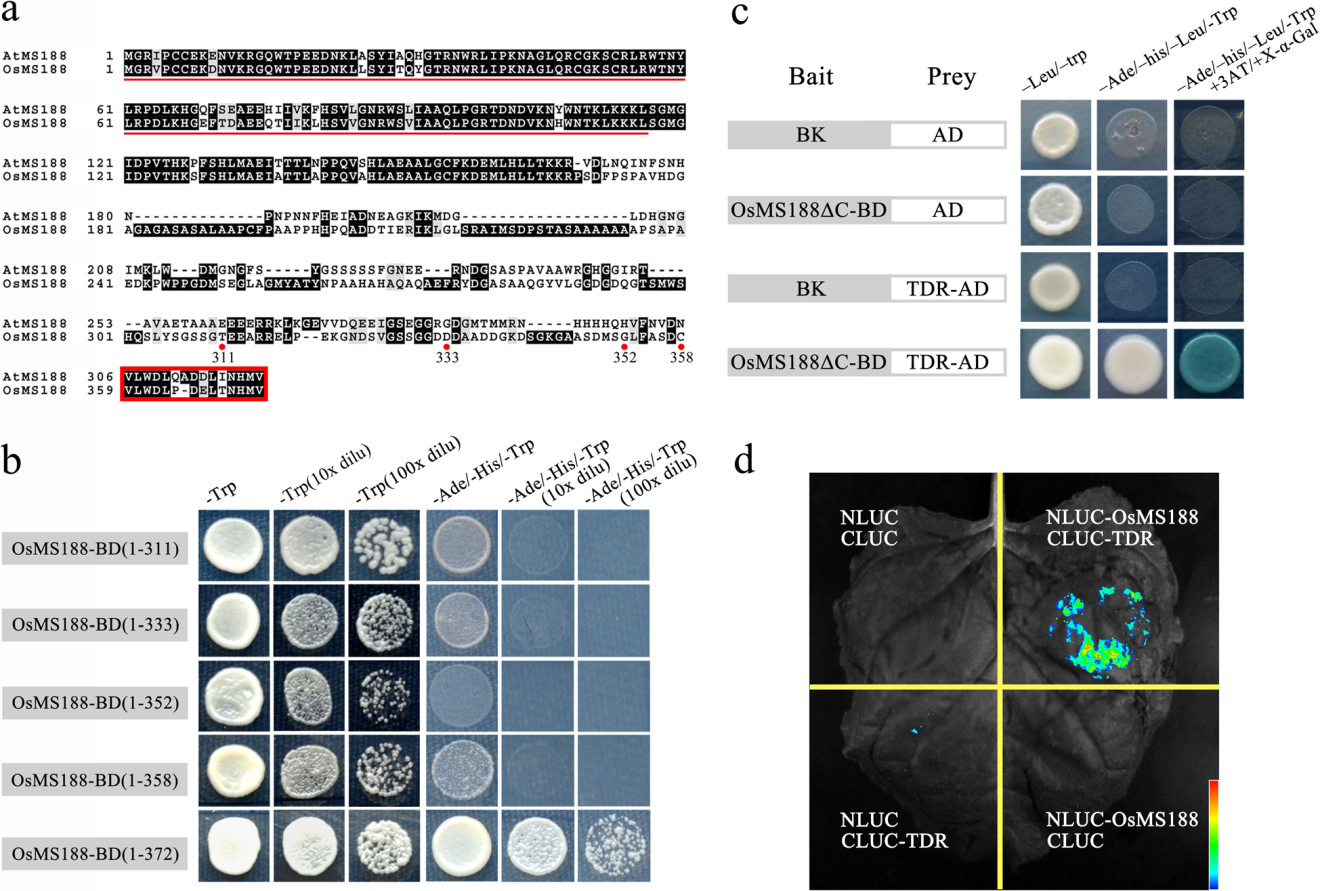
OsMS188 interacts with TDR. **a** Amino acid sequence alignment of OsMS188 and AtMS188. The red box indicates the activation domain and the red dots indicate the number of OsMS188 amino acid sequences. **b** Identification of the activation domain of OsMS188. The full-length CDS of OsMS188 allowed growth on selective media lacking Leu, His and adenine. The yeast could not grow on selective media when the last 14 amino acids were deleted. **c** Yeast two-hybrid (Y2H) assay showing the physical interaction of OsMS188 with TDR. The OsMS188 CDS without the self-activation fragment (OsMS188ΔC) was fused to the DNA-binding domain (BD) in pGBKT7, and the TDR CDS was fused to the GAL4 activation domain (AD) in pGADT7. The interactions are indicated by blue-coloured yeast colonies on SD/−Ade/−His/−Trp/−Leu/X-a-Gal media. **d** LCI assay showing the interaction between OsMS188 and TDR. Fluorescence signal intensities indicate interaction activities

MYB family members frequently interact with bHLH transcription factors to regulate downstream genes during plant growth and development (Koes et al. 2005; Appelhagen et al. 2011). Protein interaction assays were subsequently performed to analyse whether TDR interacts with OsMS188. A previous study showed that the C-terminus of AtMS188 contains an activation domain (Xiong et al. 2016). We generated several constructs with truncations of different lengths of the C-terminal region to identify the activation domain in OsMS188 (Fig. 6a). Yeast two-hybrid assays showed that AH109 strains with the full-length CDS of OsMS188 could grow on selective media lacking Leu, His and adenine. However, when the last 14 amino acids were deleted, the yeast could not grow on selective media, indicating that the activation domain of *OsMS188* was located within those 14 amino acids at the C-terminus (Fig. 6a and b). We subsequently generated a construct containing the OsMS188 CDS without this fragment (14 residues at the C-terminus) (BK–OsMS188∆C) to analyse whether it could interact with TDR. The results showed that only the yeast containing AD–TDR and BK–OsMS188∆C was able to grow on SD^–Leu/−Trp/−His/−ade^ selective media (Fig. 6c). These results indicated the existence of protein-protein interactions between OsMS188 and TDR. The interaction between these two proteins was further validated using a firefly luciferase complementation imaging (LCI) assay in tobacco. *OsMS188* and *TDR* were fused to the N-terminal (NLUC) and C-terminal (CLUC) domains of LUCIFERASE, respectively. The results showed that the cotransfection of OsMS188-NLUC together with TDR-CLUC produced strong luciferase activity, while the infiltration of the individual OsMS188-NLUC/TDR-CLUC vectors together with the corresponding empty construct failed to produce a visible signal (Fig. 6d). Both the yeast two-hybrid and LCI assays suggested interactions occur between OsMS188 and TDR.

### *OsMS188* Regulates the Expression of Several Sporopollenin Synthesis-Related Genes

**Fig. 7:**
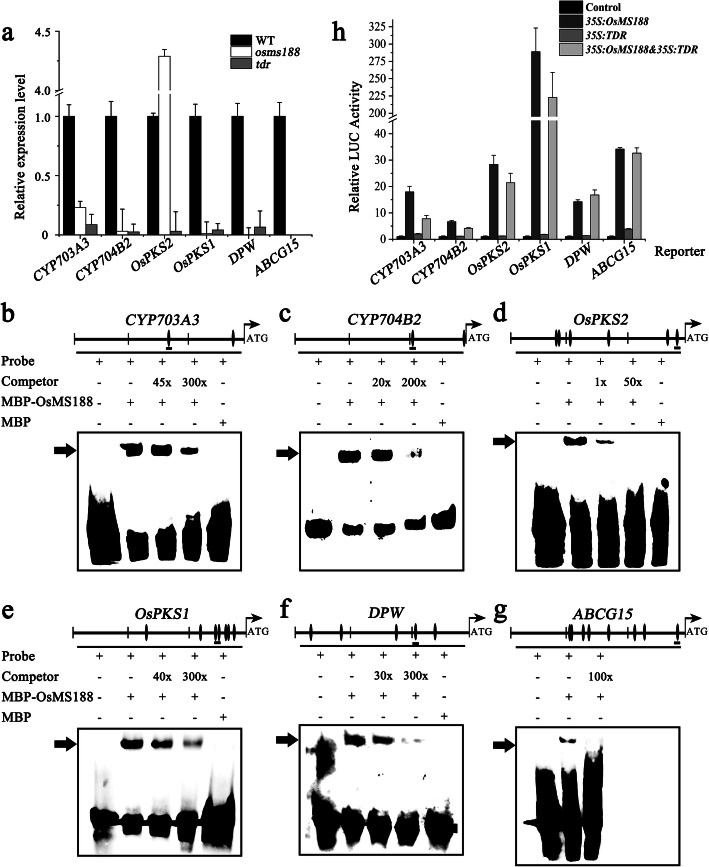
OsMS188 directly regulates the expression of *CYP703A3*, *CYP704B2*, *OsPKS2*, *OsPKS1*, *DPW* and *ABCG15*. **a** QRT-PCR analysis of *CYP703A3*, *CYP704B2*, *OsPKS2*, *OsPKS1*, *DPW* and *ABCG15* expression in the inflorescences in WT, *osms188* and *tdr* backgrounds. **b**-**g** EMSAs showing that OsMS188 can bind to the fragments of the *CYP703A3, CYP704B2*, *OsPKS2*, *OsPKS1*, *DPW and ABCG15* promoter regions. MBP-tagged proteins were mixed with a biotin-labelled probe, and unlabelled probes were used as competitors. The arrowhead indicates a shifted band. **h** Transient dual-luciferase assays were performed in Arabidopsis leaf protoplasts. A *p35S::NOS* vector was used as a negative control. *p35s::OsMS188nos* and *p35s::TDRnos* were transformed into protoplasts together with reporter plasmids. Three replicates were assessed, and the Y-axis shows the ratio of luciferase/Renilla. The errors bars indicate the SDs

*OsPKS1*, *OsPKS2*, *DPW*, *DPW2*, *DPW3*, *CYP703A3*, *CYP704B2* and *TKPR1* have been reported to be involved in sporopollenin synthesis in rice (Li et al. 2010; Shi et al. 2011; Yang et al. 2014; Xu et al. 2017; Zou et al. 2017; Shi et al. 2018; Zou et al. 2018; Xu et al. 2019; Mondol et al. 2020). Moreover, *ABCG15* and *OsABCG26* are involved in sporopollenin precursor transport (Qin et al. 2013; Wu et al. 2014; Zhao et al. 2015). QRT-PCR analyses showed that the expression of *OsPKS1*, *DPW*, *CYP703A3*, *CYP704B2*, *TKPR1*, *ABCG15* and *OsABCG26* was severely downregulated in the *osms188* mutant; however, the expression of *OsPKS2* increased 4.5-fold in the *osms188* mutant (Fig. 7a; Fig. S3). In the *tdr* mutants, the expression of all these genes was also downregulated (Fig. 7a). As OsMS188 is a member of the MYB transcription factor family, the core motif of MYB cis-elements (AACC) is present in the promoter region of the above genes. We selected *CYP703A3*, *CYP704B2*, *OsPKS1*, *OsPKS2*, *DPW* and *ABCG15* to analyse their relationship with OsMS188. EMSAs involving the purified recombinant MBP-OsMS188 proteins (Fig. S4) showed that OsMS188 could bind to specific probes containing the AACC sequences in these genes, and the shifted band was negatively correlated with the different concentrations of the competitive probe (Fig. 7b, c, d, e, f and g).

In addition, we assessed whether tapetal transcription factors could activate the expression of these sporopollenin-related genes through a protoplast transient expression system. We generated *p35s::OsMS188nos* and *p35s::TDRnos* as effectors and *pCYP703A3-LUC*, *pCYP704B2-LUC*, *pOsPKS1-LUC*, *pOsPKS2-LUC*, *pDPW-LUC* and *pABCG15-LUC* as reporters and cotransformed them into Arabidopsis protoplasts. The results showed a weak background fluorescence signal in each control group. When reporter constructs with *p35s::TDRnos* were cotransformed, LUC activity was hardly induced. However, the amount of fluorescence significantly increased when *p35s::OsMS188nos* together with these reporters was added. For the cotransformation of the reporter with both *p35s::OsMS188nos* and *p35s::TDRnos*, the LUC activity did not obviously increase (Fig. 7h). The results demonstrate that OsMS188 not only directly binds to the MYB regulatory elements of the *CYP703A3*, *CYP704B2*, *OsPKS1*, *OsPKS2*, *DPW* and *ABCG15* promoters but also activates the expression of these genes.

## Discussion

### *OsMS188* Plays Multiple Roles in Anther Development in Rice

A previous investigation showed that the downregulation of *OsMS188* via an RNAi approach leads to reduced male fertility of transgenic plants (Zhang et al. 2010). In this study, we further characterized the detailed functions of *OsMS188* in anther development and pollen formation through knock-out mutant analysis. We used the CRISPR-Cas9 technique to generate two complete male sterility-inducing alleles of the *OsMS188* gene with a frame-shift mutation (Fig. 1). Cytological observations showed that the tapetal cells of *osms188* exhibit an abnormal development phenotype with aberrant vacuolization (Fig. 2). Recently, a knockout line of *OsMS188* was reported to exhibit premature tapetal cell death (Pan et al. 2020). Therefore, OsMS188/OsMYB80 is an essential regulator of tapetum development in rice. During anther development, the tapetum undergoes endomitosis and gradual apoptosis/programmed cell death (PCD) to complete its development and fulfil its function (Stevens and Murray 1981; Vizcay-Barrena and Wilson 2006). TUNEL assays showed that a weak fluorescence signal was detected in aberrant tapetal cells of the *osms188* mutant, suggesting that OsMS188 is involved in tapetal PCD progression (Fig. 3). PTC1 has been reported to control programmed tapetum development in rice (Li et al. 2011) and to act as a downstream target of OsMS188 (Pan et al. 2020). Therefore, OsMS188 is likely to regulate tapetum programmed death through PTC1 during anther development.

The pollen wall protects the developing microspore through its resistance to external stresses and promotion of the identification and attachment of pollen to thestigma (Ariizumi et al. 2004). The cuticle layer on the anther surface plays an important role in protecting organisms against water loss, UV irradiation, and frost damage (Jung et al. 2006). In rice, sexine formation depends on the synthesis and modification of lipids, and the wax layer is composed of very-long-chain fatty acids. In rice, mutants of most pollen wall-related genes, including *CYP703A3*, *CYP704B2*, *OsPKS1*, *OsPKS2*, *DPW*, *ABCG15*, *OsABCG26*, *DPW2*, *DPW3* and *TKPR1,* exhibit not only defective exine deposition but also abnormal formation of the wax layer on the anther surface (Li et al. 2010; Shi et al. 2011; Qin et al. 2013; Yang et al. 2014; Zhao et al. 2015; Zhang et al. 2016; Xu et al. 2017; Zou et al. 2017; Shi et al. 2018; Zou et al. 2018; Xu et al. 2019; Mondol et al. 2020). In *osms188*, both the sexine layer around the pollen grains and the wax layer on the anther surface were absent (Fig. 2). Previous studies showed that the expression of *CYP703A3* was downregulated in *tdr* and *gamyb* mutants. TDR and GAMYB can bind to the promoter of CYP703A3 (Yang et al. 2014; Aya et al. 2009). However, it is not clear whether they can activate *CYP703A3* expression. In this work, gene expression analysis showed that the expression of all of the above pollen wall-related genes was downregulated in *osms188* anthers, suggesting that they act downstream of *OsMS188* (Fig. 7a; Fig. S3). EMSAs and protoplast dual-luciferase assays showed that OsMS188 not only directly bound to the promoters of *CYP703A3*, *CYP704B2*, *OsPKS1*, *OsPKS2*, *DPW* and *ABCG15* but also activated their expression as a main-effect factor during pollen wall formation (Fig. 7). Considering the absence of the exine and cuticle layer in *osms188*, we propose that the transcriptional regulatory pathway for lipid synthesis in the tapetum is also shared with exine and anther cuticle formation in rice, although the transport mechanism remains unclear. Therefore, the sporopollenin synthesis pathway might be responsible for the formation of the pollen wall and anther cuticle at the same time in rice. *OsMS188* plays multiple roles during anther development, including tapetum development, pollen wall formation and anther surface formation.

### The Transcriptional Regulatory Pathway of Rice Tapetum Development

**Fig. 8:**
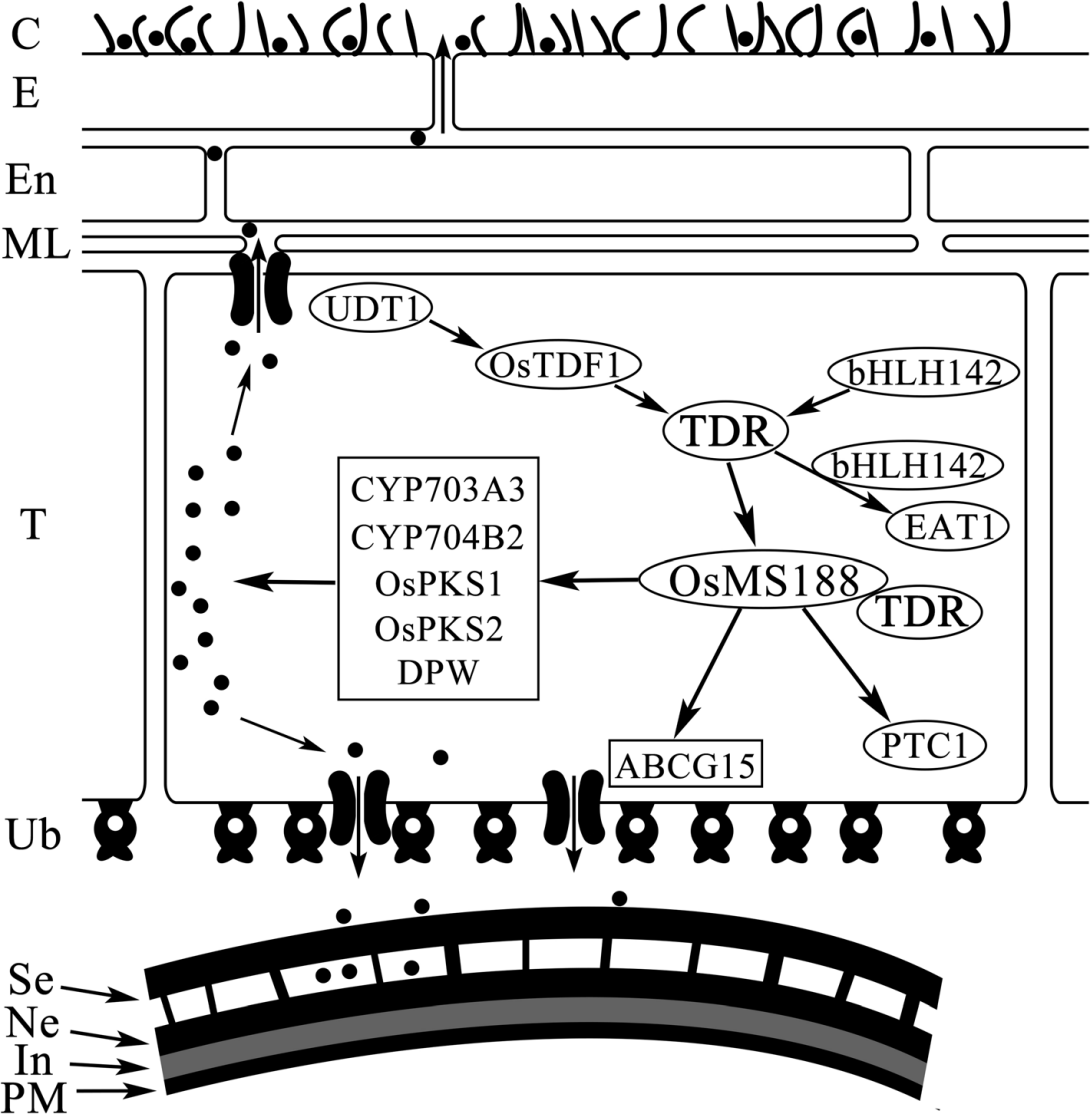
Proposed model for rice tapetum-related gene regulatory network. In the genetic pathway of the rice tapetal layer, the oval circles represent transcription factors. The genes in the box represent downstream enzymes. The black arrows represent positive regulation, and the black dots represent lipid synthesis in tapetal cells. This model indicates that *OsMS188* acts as a core hub to modulate tapetum development and pollen wall and cuticle formation. C, cuticle; E, epidermis; En, endothecium; In, intine; ML, middle layer; Ne, nexine; PM, plasma membrane; Se, sexine; T, tapetum; Ub, Ubisch body

In rice, several transcription factors regulating tapetum development have been reported, including *UDT1*, *OsTDF1*, *bHLH142/TIP2*, *TDR*, *EAT1* and *PTC1* (Jung et al. 2005; Li et al. 2006; Li et al. 2011; Niu et al. 2013; Fu et al. 2014; Ko et al. 2014; Cai et al. 2015). The ablation of *UDT1*, *OsTDF1*, *TDR* and *TIP2* leads to vacuolated and hypertrophic tapetal cells, suggesting that these genes are involved in early tapetum development (Jung et al. 2005; Li et al. 2006; Niu et al. 2013; Fu et al. 2014; Cai et al. 2015). In this study, we showed that the MYB transcription factor OsMS188 is strongly expressed in tapetal cells and acts as an essential regulator of their development and degradation (Fig. 3; Fig. 4). A previous study proposed a regulatory relationship among TDR, bHLH142 and EAT1 and suggested that these proteins regulate the downstream genes *CYP703A3*, *CYP704B2* and *OsC6* (Shi et al. 2015). This work and other studies (Cai et al. 2015; Pan et al. 2020) further expand the known regulatory network of tapetum development and functions (Fig. 8). This network includes two additional regulators: OsTDF1 and OsMS188. *OsTDF1* acts downstream of *UDT1* and upstream of *TDR* and *EAT1* (Cai et al. 2015). Among these upstream regulators, TDR directly regulates *OsMS188* (Fig. 5; Fig. S2), and OsMS188 directly regulates sporopollenin-related genes for pollen wall formation (Fig. 7). MYB family members frequently interact with bHLH transcription factors to regulate downstream gene expression (Koes et al. 2005; Appelhagen et al. 2011). Both Y2H assays and EMSAs suggested that TDR interacted with OsMS188 to regulate downstream gene expression (Fig. 5; Fig. 6). Although TDR could bind to the promoter of sporopollenin related genes (Yang et al. 2014; Shi et al. 2015), transient dual-luciferase assays showed that TDR is unlikely to directly activate the expression of these genes (Fig. 7h). We speculate that TDR regulates pollen wall formation by activating the expression of *OsMS188* during anther development. Additionally, GAMYB and PTC2 have also been reported to regulate exine formation and PCD of tapetal cells in rice anther development (Aya et al. 2009; Uzair et al. 2020). PTC2 is the homologue of TEK, an AT-hook motif nuclear-localized protein essential for nexine formation in Arabidopsis. AMS (an orthologue of TDR) directly regulates *TEK* and *MS188* expression for sexine and nexine formation (Lou et al. 2014). We propose that *PTC2* and *OsMS188* are parallel in the rice tapetal genetic pathway regulated by TDR. However, our Y2H assays showed that they also did not interact with OsMS188 (Fig. S5). PTC1 is a regulator of late tapetum development, as the tapetum of *ptc1* is aberrantly degenerated after meiosis (Li et al. 2011), and *PTC1* acts downstream of OsMS188 (Pan et al. 2020). Combined with the regulatory role of OsMS188 in the activation of sporopollenin synthesis, OsMS188 plays a central role in tapetum development, pollen formation and anther cuticle formation (Fig. 8). All the genes in this network are essential for anther development and pollen formation. Plants with mutations in these genes exhibit a male-sterile phenotype. This gene regulatory network will be helpful forfuture investigation of anther development as well as fertility regulation in rice.

## Materials and Methods

### Plant Materials and Growth Conditions

All the rice plants used in this study were grown in the botanical garden at Shanghai Normal University (Shanghai, China). The generation of the *osms188–1* and *osms188–2* mutants was mediated by CRISPR-Cas9 technology. The target sites in the *osms188* coding sequence were identified with designer software and fused into the CRISPR-Cas9 plasmids. The constructed plasmids were subsequently introduced into *Agrobacterium tumefaciens* EHA105, which were then transformed into wild type Nipponbare rice.

### Phenotypic Analysis of the Mutant

The plants were imaged with a Nikon D7000 digital camera (Nikon, Japan) and an Olympus SZX10 dissecting microscope (Olympus, Japan). Images of spikelets and anthers were captured with an Olympus BX51 fluorescence microscope (Olympus, Japan). Pollen viability was assessed using Alexander’s solution for staining wild-type and mutant anthers. By the use of semi-thin sections along with SEM (JEOL, Japan) and TEM (Hitachi, Japan) observations, the spikelets and anthers of wild type and *osms188* mutant plants were classified as belonging to different stages to avoid experimental deviation. The embedding and observation procedures were performed as described in a previous study (Lou et al. 2014).

### TUNEL Assay

Wild type and *osms188* spikelets were fixed in FAA solution for 1 day at 4 °C. The samples were then dehydrated through an ethanol gradient and xylene and embedded in paraffin (Sigma, USA). The embedding block was sectioned to a thickness of 7 μm by an MR2 rotary microtome (RMC, USA) attached to Poly-Prep slides (Sigma, USA). The TUNEL apoptosis detection process was performed with a DeadEnd Fluorometric TUNEL system kit (Promega, USA) according to the product’s instructions. Images were obtained using an Olympus FV3000 laser scanning microscope (Olympus, Japan).

### RNA In Situ Hybridization

The embedding block of wild type spikelets was sectioned to a thickness of 8 mm using an MR2 rotary microtome (RMC, USA). A 415-bp specific fragment of the *OsMS188* CDS was cloned into a pBluescript-SK vector (Stratagene, USA). Plasmid DNA was completely digested using EcoRI or HindIII. The antisense and sense probes of OsMS188 were transcribed using a digoxigenin (DIG) RNA labelling kit (Roche, Switzerland) according to the product’s instructions. RNA hybridization and immunological detection of the hybridized probes were performed as described (Zhu et al. 2011, Shi et al. 2018). Afterward, the samples were imaged via an Olympus DP73 digital camera (Olympus, Japan).

### RT-PCR and qRT-PCR

Total RNA was isolated from the spikelets of wild-type and *osms188* plants at different stages using a TRIzol kit (Invitrogen, USA). Reverse transcription was performed using TransScript Fly First-Strand cDNA Synthesis SuperMix (TransGen Biotech, China). The semiquantitative RT-PCR procedure was performed as described previously (Zhang et al. 2007). QRT-PCR analyses of each sample were performed in conjunction with SYBR Green Real-time PCR Master Mix (Toyobo, Japan) and an ABI 7300 system (Life Technologies, USA). The quantitative PCR procedure and conditions were the same as those previously described (Xiong et al. 2016). *Actin* was used as an internal control. Three biological replicates were performed for each experiment. The sequences of all the primers used are listed in Table S1.

### Electrophoretic Mobility Shift Assays (EMSAs)

The full-length CDSs of OsMS188, TDR, bHLH142 and EAT1 were cloned into a pMAL-c5X vector (GE Healthcare, USA) to generate the MBP-*OsMS188*, MBP-*TDR*, MBP-*bHLH142* and MBP-*EAT1* constructs. The expression and purification of the fusion proteins were conducted according to the manufacturer’s instructions. Labelled or unlabelled probes containing the core motifs of the *OsMS188* binding sites of the *CYP703A3*, *CYP704B2*, *OsPKS1*, *OsPKS2*, *DPW and ABCG15* promoters were generated by using specific primers (Table S1), and probes were also generated for *TDR* in *OsMS188.* A LightShift Chemiluminescent EMSA Kit (Thermo Scientific, USA) was used to perform the EMSAs. The resulting images were captured with a Tanon-5500 Chemiluminescent Imaging System.

### Dual-Luciferase Transient Expression Assays of Arabidopsis Protoplasts

Protoplasts obtained from Arabidopsis (Col-0) leaves were grown for 21–28 day and digested with 0.75% (w/v) cellulase R10 and 0.175% (w/v) macerozyme R10 (Yakult Honsha, Tokyo). The plasmids *p35S::TDR-nos* and *p35S::OsMS188CDS-nos* and the pGreenII 0800-LUC vector containing the promoters of *OsMS188*, *CYP703A3*, *CYP704B2*, *OsPKS1*, *OsPKS2*, *DPW and ABCG15* were cotransformed into protoplasts mediated by 40% (w/v) PEG4000 and cultivated overnight. After lysing the protoplasts by the addition of passive lysis buffer, firefly and Renilla luciferase activities were quantified using a Dual-Luciferase Reporter Assay System (Promega, USA) and detected with a GloMax Navigator Microplate Luminometer (Promega, USA), according to the manufacturers’ instructions.

### Phylogenetic Analysis

The homologous protein of OsMS188 in *Arabidopsis thaliana* was identified using the Basic Local Alignment Search Tool (BLAST) of the National Center for Biotechnology Information (http://www.ncbi.nlm.nih.gov/). Multiple sequence alignments of the full-length protein sequences were performed using Clustal W and displayed using BoxShade (http://www.ch.embnet.org/software/ClustalW.html).

### Yeast Two-Hybrid Assays

The yeast two-hybrid assays were performed following the protocol of a Clontech two-hybrid system (Clontech, USA). The CDSs of OsMS188 (different lengths) were amplified and fused into pGBKT7 plasmids to determine the self-activation domain. Similarly, the coding sequences of *TDR*, *GAMYB* and *PTC2* were inserted into pGADT7 plasmids. These constructs were co-transformed into the AH109 yeast strain that was screened under selective growth conditions as described in a previous study (Xiong et al. 2016).

### LCI Assays

The coding sequences of *OsMS188* and *OsTDR* were cloned into JW771-NLUC and JW772-CLUC, respectively. LCI assays were performed as described in previous studies (Zhang et al. 2015; Wang et al. 2019). *Agrobacterium tumefaciens* (strain GV3101 with pSoup-p19 vector) containing the recombinant plasmid was infiltrated into *Nicotiana benthamiana* leaves via needleless syringes.

## Supplementary Information

**Additional file 1: Table S1.** Sequences of primers used in this study.

**Additional file 2: Fig. S1.** Semi-thin sections of the wild type and *osms188* mutant. Bars = 20 μm.

**Additional file 3: Fig. S2.** RT-PCR analysis of the expression of *OsMS188* in inflorescences of WT, *udt1*, *ostdf1*, *tdr*, *bhlh142* and *eat1* after 30 and 35 cycles. gDNA: genomic DNA.

**Additional file 4: Fig. S3.** QRT-PCR analysis of the expression of *DPW2*, *DPW3*, *TKPR1* and *OsABCG26* in inflorescences of the *osms188* mutant.

**Additional file 5: Fig. S4.** The OsMS188 protein was expressed and purified from Rosetta *Escherichia coli*. The black box indicates the target band.

**Additional file 6: Fig. S5.** Y2H assay showing that OsMS188 cannot interact with GAMYB or PTC2.

## Data Availability

All data generated or analyzed during this study are included in this published article and its supplementary information files.

## Abbreviations

EMSAs: Electrophoretic mobility shift assays
LCI: Luciferase complementation imaging
LUC: Luciferase
MBP: Maltose binding protein
qRT-PCR: Quantitative real-time PCR
RT-PCR: Reverse transcription-PCR
SEM: Scanning electron microscopy
TEM: Transmission electron microscopy
TUNEL: Terminal deoxynucleotidyl transferase–mediated dUTP nick-end labelling
WT: Wild-type
Y2H: Yeast two-hybrid
