# Supplementary figures and images for: OsMS188 Is a Key Regulator of Tapetum Development and Sporopollenin Synthesis in Rice
Source: Rice (N Y). 2021 Jan 6;14:4. doi: 10.1186/s12284-020-00451-y (PMC7788135; doi:10.1186/s12284-020-00451-y)

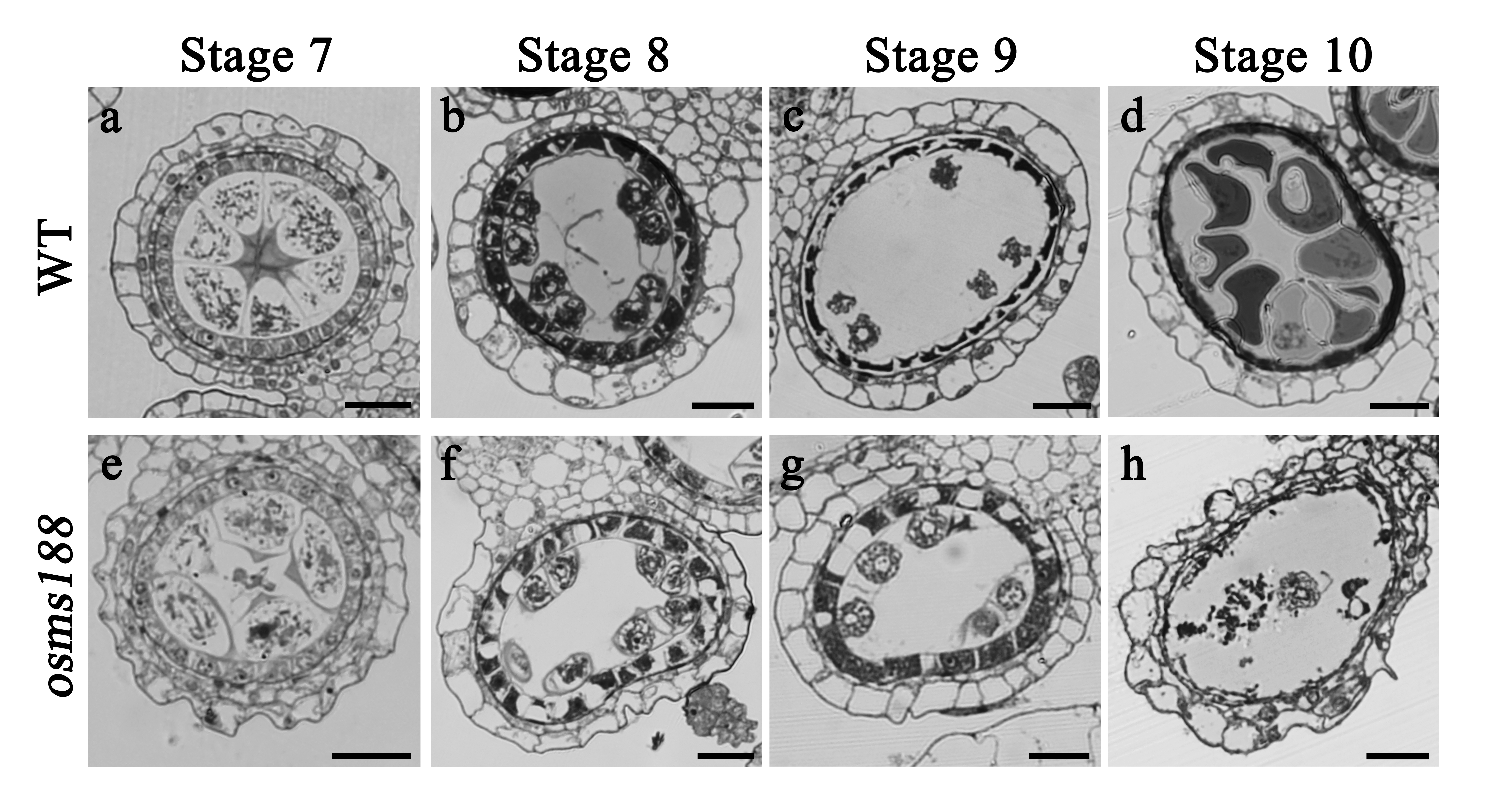

Supplement: Supplementary file 2 — Additional file 2: Fig. S1. Semi-thin sections of the wild type and osms188 mutant. Bars = 20 μm. [file 12284_2020_451_MOESM2_ESM.jpg]

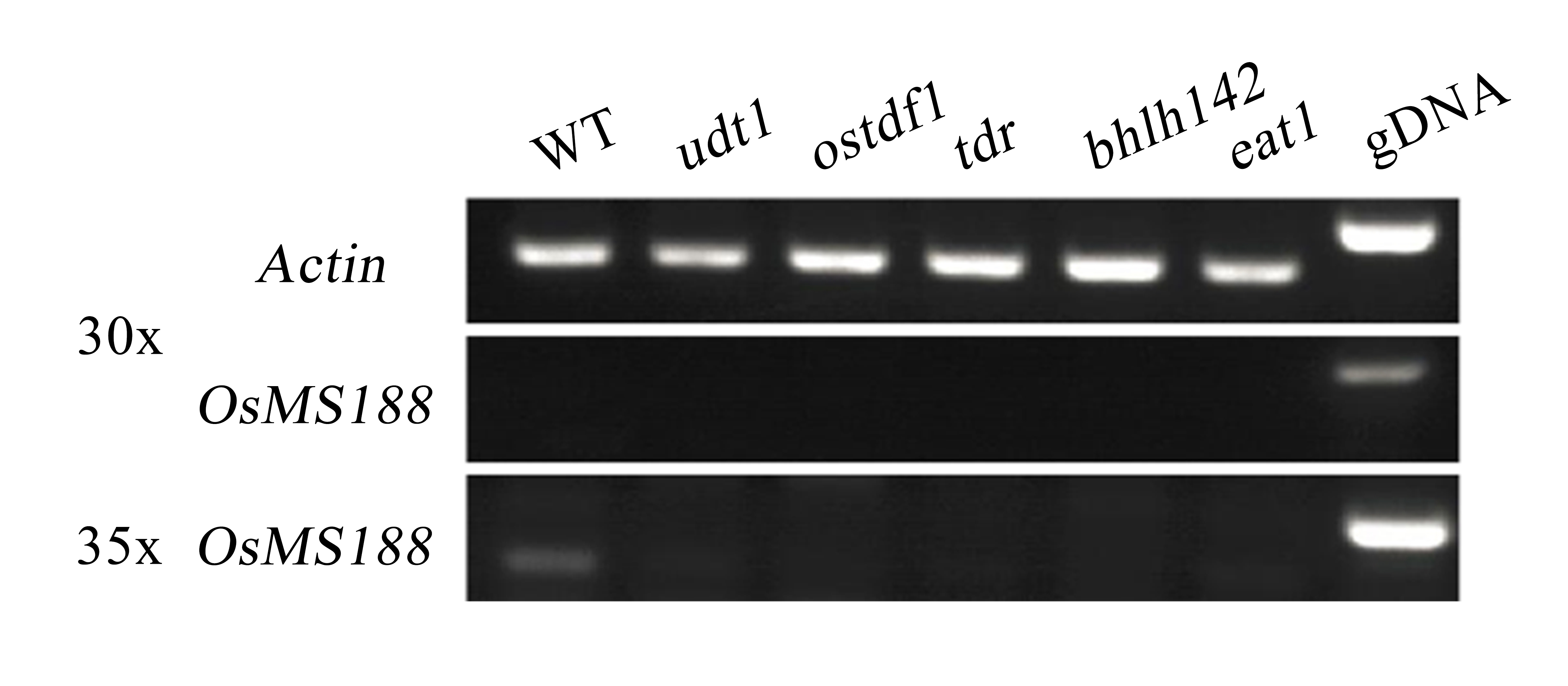

Supplement: Supplementary file 3 — Additional file 3: Fig. S2. RT-PCR analysis of the expression of OsMS188 in inflorescences of WT, udt1, ostdf1, tdr, bhlh142 and eat1 after 30 and 35 cycles. gDNA: genomic DNA. [file 12284_2020_451_MOESM3_ESM.jpg]

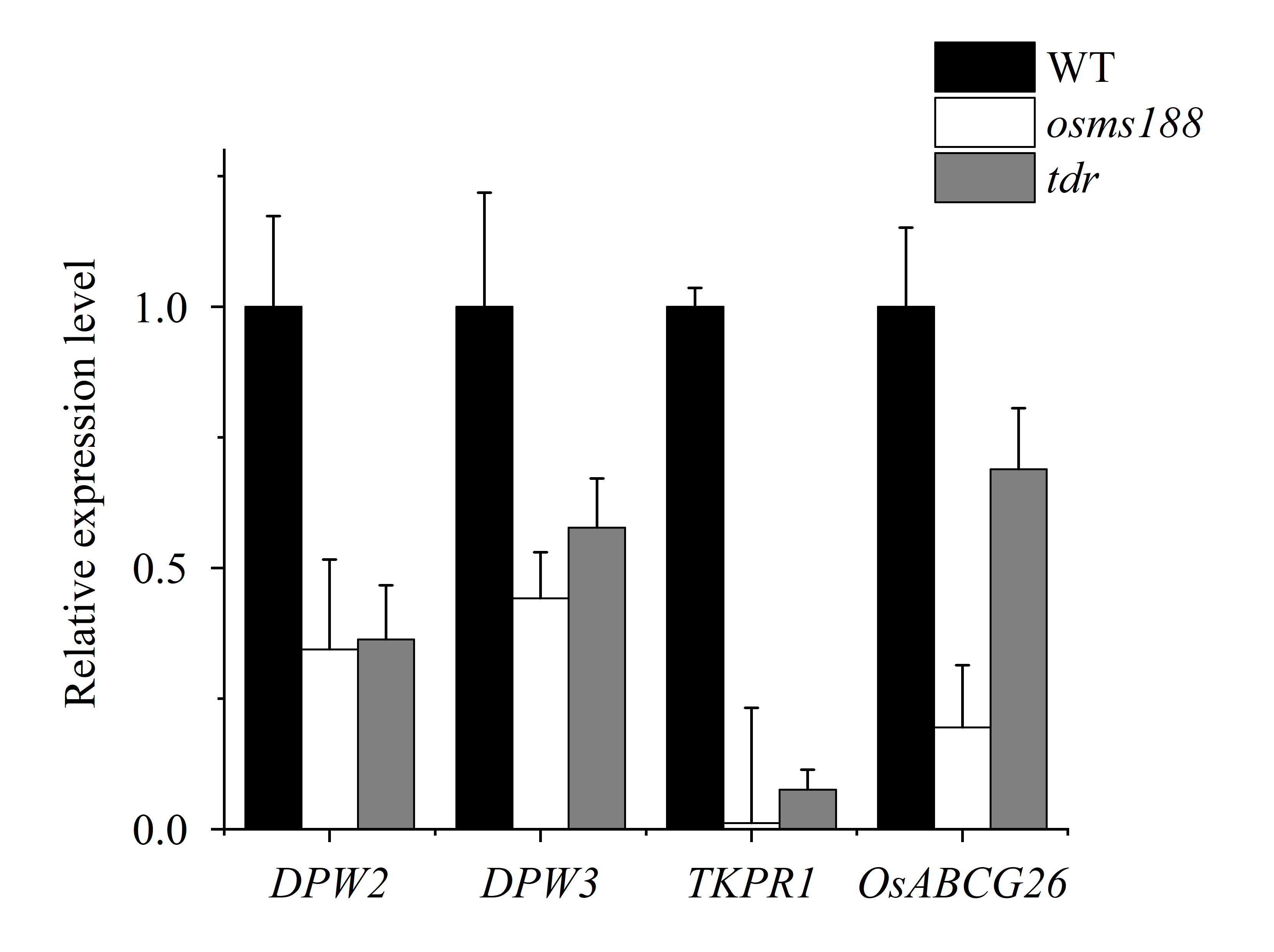

Supplement: Supplementary file 4 — Additional file 4: Fig. S3. QRT-PCR analysis of the expression of DPW2, DPW3, TKPR1 and OsABCG26 in inflorescences of the osms188 mutant. [file 12284_2020_451_MOESM4_ESM.jpg]

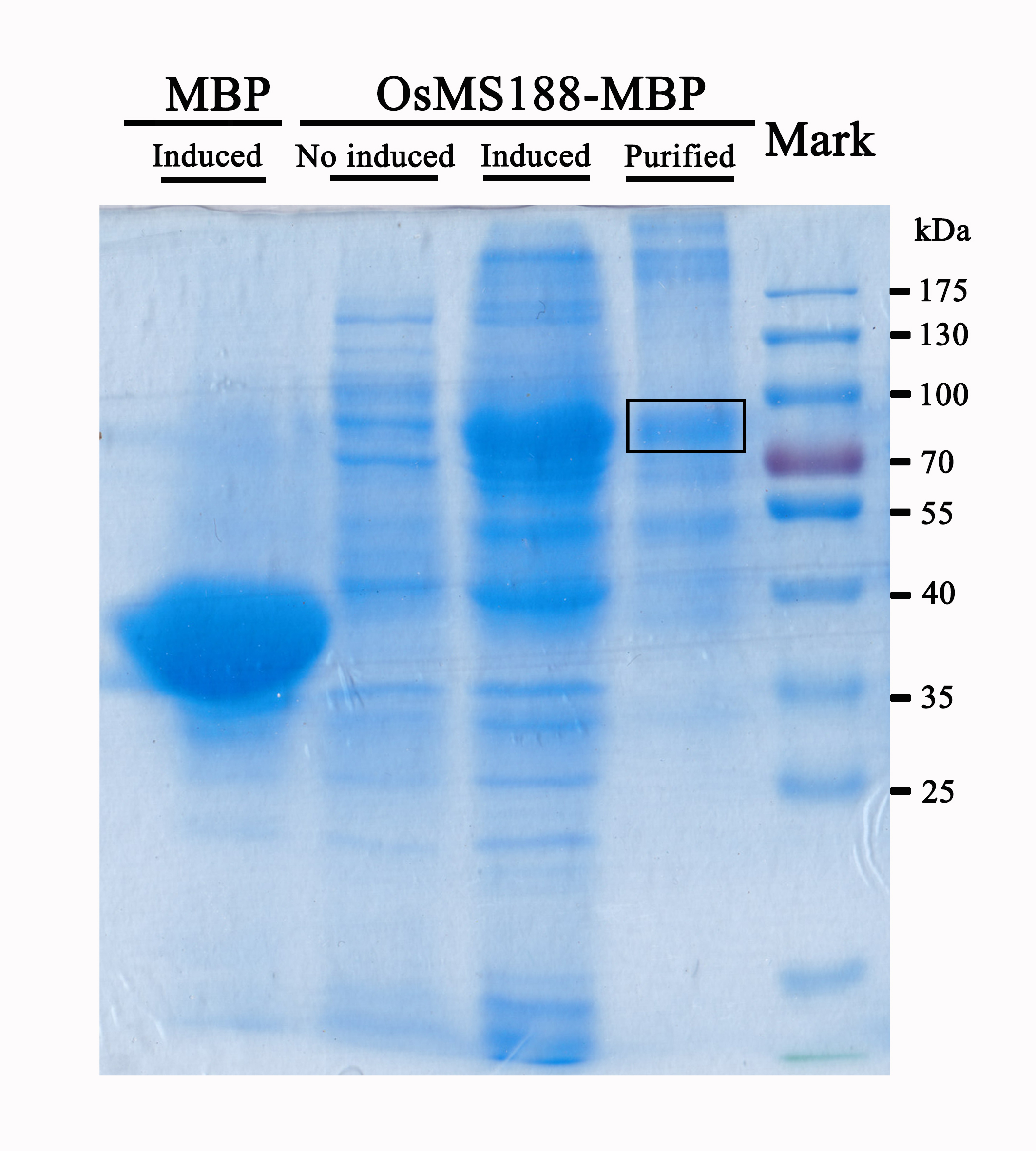

Supplement: Supplementary file 5 — Additional file 5: Fig. S4. The OsMS188 protein was expressed and purified from Rosetta Escherichia coli. The black box indicates the target band. [file 12284_2020_451_MOESM5_ESM.jpg]

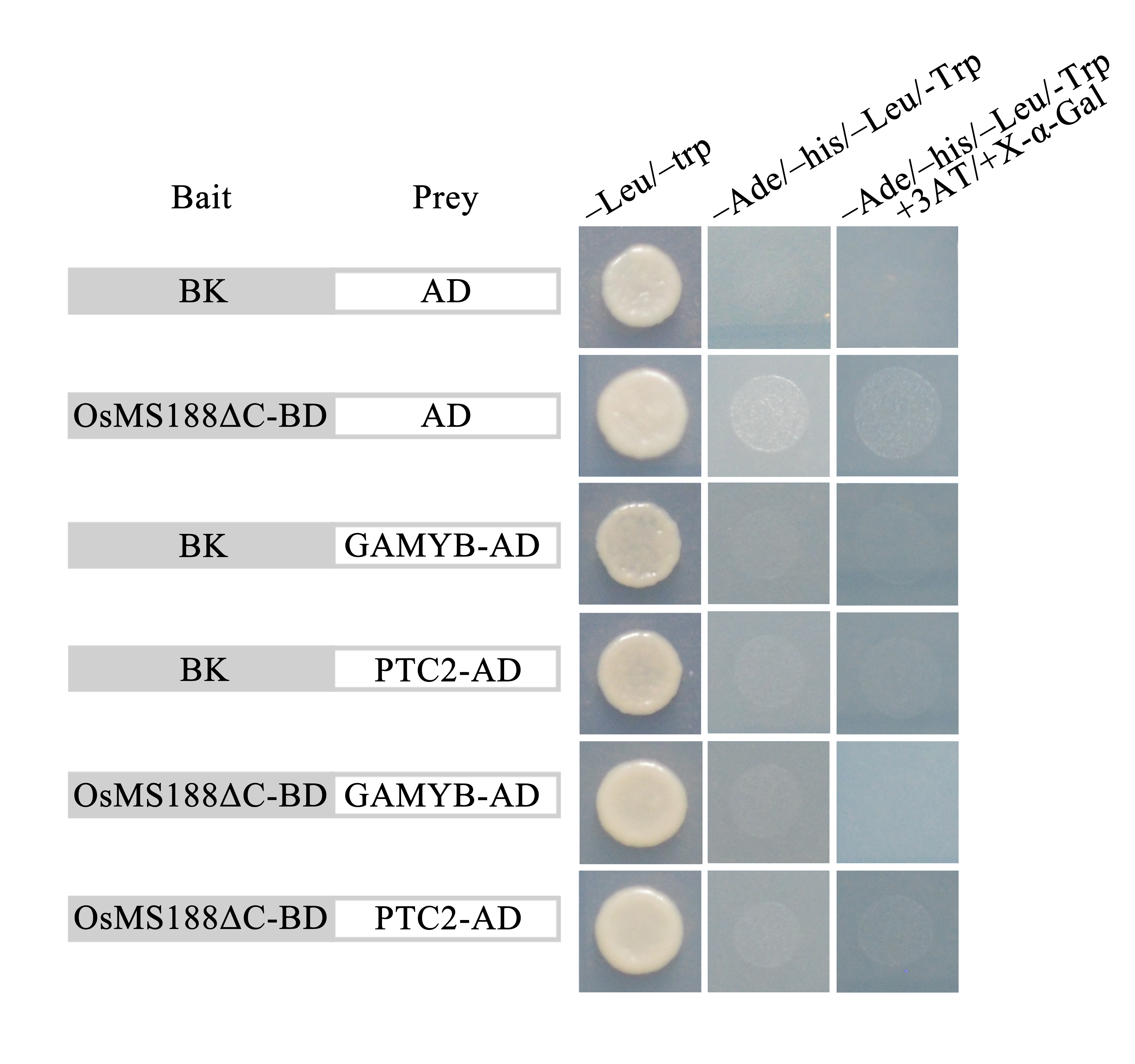

Supplement: Supplementary file 6 — Additional file 6: Fig. S5. Y2H assay showing that OsMS188 cannot interact with GAMYB or PTC2. [file 12284_2020_451_MOESM6_ESM.jpg]
